# Inhaled Surfactant in the treatment of accidental Talc Powder inhalation: a new case report

**DOI:** 10.1186/1824-7288-37-47

**Published:** 2011-09-27

**Authors:** Federico Matina, Mirella Collura, Maria Cristina Maggio, Patrizio Vitulo, Caterina Lo Piparo, Giovanni Corsello

**Affiliations:** 1 Clinica Pediatrica. Dipartimento Universitario Materno-Infantile. Università degli Studi di Palermo. Via Alfonso Giordano, 3. Palermo. 90143. Italy; 2 Cystic Fibrosis Center. ARNAS Civico G. Di Cristina Hospital. Via dei Benedettini, 1. Palermo. 90100. Italy; 3 Respiratory Medicine ISMETT UPMC. Via Ernesto Tricomi, 1. Palermo. 90127. Italy

**Keywords:** Talcum powder, Accidental Inhalation, Respiratory Distress, Surfactant, Bronchopulmonary Lavage, Respiratory Physiotherapy

## Abstract

The use of talcum powder is incorrectly part of the traditional care of infants. Its acute aspiration is a very dangerous condition in childhood. Although the use of baby powder has been discouraged from many authors and the reports of its accidental inhalation have been ever more rare, sometimes new cases with several fatalities have been reported. We report on a patient in which accidental inhalation of baby powder induced severe respiratory difficulties. We also point out the benefits of surfactant administration. Surfactant contributed to the rapid improvement of the medical and radiological condition, preventing severe early and late complications and avoiding invasive approaches.

## Introduction

The use of talcum powder is incorrectly part of the traditional care of infants. Acute aspiration of baby powder is a very dangerous condition in childhood and several fatalities have been reported [1]. Talcum Powder consist of about 90% talc (anhydrous magnesium silicate), the remainder being mainly magnesium carbonate or zinc oxide according to the preparation [2]. Many inhalation accidents have been reported to have occurred during a nappy change [3,4]. There is nearly always a characteristic silent period of several hours between the initial event and the beginning of severe respiratory distress. This asymptomatic period can lead to wrong medical decisions. The respiratory distress is due mainly to bronchiolar obstruction by the aspirate powder and may evolve to a massive bronchitis and bronchiolitis with pulmonary oedema, atelectasis, and compensatory emphysema [1]. Reports about accidental inhalation of talcum powder are relatively rare, but despite its use has been discouraged by many authors, some new cases have been recently reported [5]. We report on a patient in whom the accidental inhalation of baby powder induced severe respiratory difficulties. She was treated with surfactant administration showing full clinical recovery.

## Case Report

**Figure 1:**
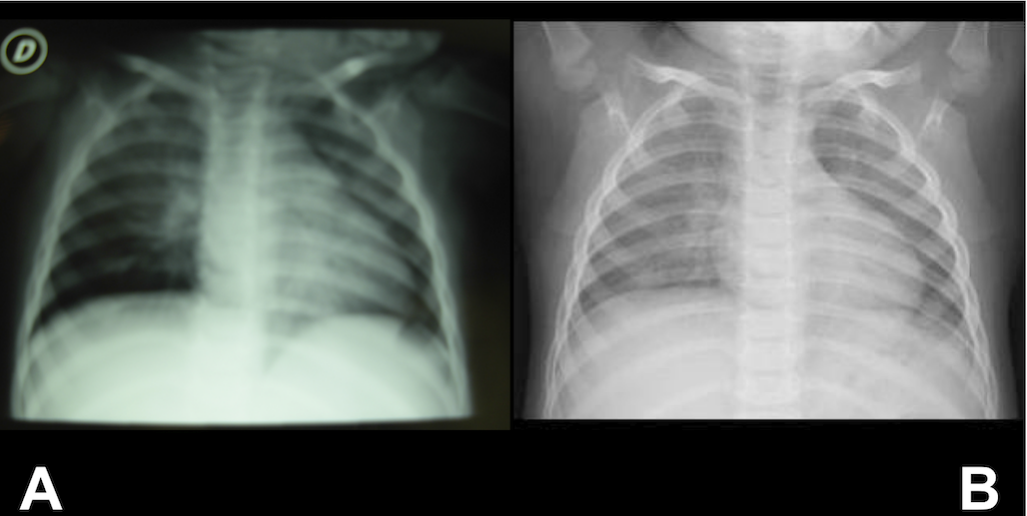
**Chest x-ray on admission (*a*) and five days after (*b*)**.

A18 month female child was admitted for appearance of cough and respiratory distress after one hour from the inhalation of talc powder, accidentally happened during a nappy change. She was apyrectic and presented with cough and only a mild respiratory distress, tachypnea (40/minute), 98% oxygen saturation, heart frequency of 145/minute. On examination there was no evidence of cyanosis, the respiratory sounds were normal on both sides and other systems were normal. Chest x-ray revealed a consolidation in central and middle lung zones and basal emphysema on both sides (Figure 1a). An empirical treatment with intravenous antibiotic (Ceftriaxone at the dose of 50 mg/Kg) and steroids (Methylprednisolone at the dose of 1 mg/Kg/8 h) was done for five days. A topical treatment by aerosol with surfactant (Poractant Alfa), budesonide and salbutamol was added. Surfactant was given at the dose of 120 mg/1,5 ml and it was repeated after 12 hours; after then the aerosol therapy was administered only with budesonide (0,25 mg/dose) and salbutamol (2 mg/dose). Inhalation therapy was carried out with a pneumatic nebulizer and with a spacer chamber, with aiming to reduce oropharyngeal deposition and increasing distal bronchial deposition. A respiratory physiotherapy by clapping was performed twice a day for a week. Chest X-ray showed a clear improvement of the lung condition after five days of therapy, showing only an increased perihilar interstitial marking on both sides (Figure 1b). The respiratory functions preserved normal without signs of deterioration. Coughing has reduced till complete disappearance. On clinical follow-up we found no symptoms and signs of respiratory tract or pulmonary disease. Chest x-ray examinations performed at 1,5 and 6 months resulted normal.

## Discussion

Baby powders are usually insoluble in water and may drie up the surface of the mucous membranes of the tracheobronchial tree, thus impairing the function of cilia and the mechanism of pulmonary clearance. Bronchoalveolar lavage is ineffective in washing away the talc powder from the airways, due to its water insolubility [6]. Furthermore prolonged inhalation of powder may induce a surfactant depletion, bronchiolar hyperresponsiveness and alveolar damage. In our case the administration of exogenous surfactant via aerosol was well tolerated and appeared to be safe. In fact due to its chemical-physical properties, the surfactant, by inducing the formation of micelle, may adsorb the inhaled magnesium-silicate powder and prevent its deposition on the alveolar surface, favoring its purging by expectoration. It may also replaces the endogenous surfactant, which could be seriously decreased due to the consumption by the fine powder inhalation. Exogenous surfactant therefore improves the aeration and reduces the lung damage [7]. In our experience, the surfactant administration contributed to the rapid improvement of the medical and radiological condition, preventing severe early and late complications caused by talcum powder inhalation. Possibily, treatment with respiratory physiotherapy was also helpful, particularly by means of percussions causing "pressure waves" to extend inside the thorax, ultimately taking thick secretions away from the bronchial wall. It also stimulates the muscular contractility. We proposed the use of Salbutamol and Budesonide in order to reduce bronchiolar hyperresponsiveness and alveolar damage. The bronchoalveolar lavage with saline solution, previously used for the treatment of the talc powder inhalation, could be scarcely effective because of the insolubility of talc powder in a watery solvent. One of characteristics of the inhalation of the baby powder, indeed, is that the mechanics of pulmonary clearance appear to be severely disrupted. In particular, a large surface area associated with even a modest quantity of a finely divided solid, such as baby powder, acts as a sink for the adsorption of fluids, which are taken up more readily when their surface tension is low. Thus, as Motomatsu et al [6] supposed, the nature of surface activity also suggests that the efficiency of lavage for cleaning powder from the lungs would be enhanced if a material such as dipalmitoyl-lecithin and natural surfactant were added to the fluid for lavage. Our opinion is that the present report of such patient may be useful to clarify a new therapeutic approach to patients with a rare although potentially severe condition. Other reports and larger studies are needed to determine optimal dosing and to confirm the efficacy and the safety of inhaled surfactant compared with other potentially effective therapeutic modalities.

## Consent

Written informed consent was obtained from the parents of the patient for publication of this case report and any accompanying images. A copy of the written consent is available for review by the Editor-in-Chief of this journal.

## Competing interests

The authors declare that they have no competing interests.

## Authors' contributions

FM drafted the manuscript and participated in management of the case. MC and PV managed the case and participated in design of the study. MCM participated in management of the case and in drafting the manuscript. CLP performed the respiratory physiotherapy and participated in drafting the manuscript. GC coordinated the study and participated in its design. All authors read and approved the final manuscript.
